# Experiences and views of people with diabetes during Ramadan fasting: A qualitative meta-synthesis

**DOI:** 10.1371/journal.pone.0242111

**Published:** 2020-11-23

**Authors:** Jieying Liao, Tianfang Wang, Zhan Li, Haotian Xie, Shanshan Wang

**Affiliations:** 1 School of Traditional Chinese Medicine, Beijing University of Chinese Medicine, Beijing, P. R. China; 2 Department of Endocrinology, Beijing Hospital of Traditional Chinese Medicine Huairou Branch, Beijing, P. R. China; 3 School of Humanities, Beijing University of Chinese Medicine, Beijing, P. R. China; University of Birmingham, UNITED KINGDOM

## Abstract

**Objectives:**

To review and appraise the existing qualitative studies on Ramadan fasting in participants with diabetes and to integrate valuable qualitative evidence for optimizing diabetes management.

**Methods:**

Twelve databases (PubMed, Embase, Cochrane Library, Science Direct, CINAHL, PsycINFO, JBI (Joanna Briggs institute), Web of Science, and four Chinese databases) were searched to identify qualitative studies on experiences and perspectives of Ramadan fasting in participants with diabetes. CASP (Critical Appraisal Skills Program) Qualitative Checklists were applied to appraise the included studies. A meta-synthesis approach was used to analyze the included studies. Through the strategy of inductive thematic synthesis and reciprocal interpretation, the findings and quotations of the included studies were integrated into new themes and categories. The CERQual (Confidence in the Evidence from Reviews of Qualitative Research) tool was used to grade the confidence of the new themes.

**Results:**

A total of 11 qualitative studies were included, and 43 findings were isolated. Ten new themes were identified and synthesized from the findings. Finally, four new categories were integrated, including the knowledge and understanding of observing Ramadan fasting, well-being and challenges, self-efficacy, and needs and expectations of participants with diabetes during Ramadan.

**Conclusions:**

Insulin-dependent individuals call for special concern during Ramadan fasting. Ramadan-focused education needs to be developed and generalized, and existing guidelines should be improved to optimize the management of diabetes. Professional HCPs contribute to weigh the health risks and mental satisfaction for their patients, partly, to balance health and religion. Participants’ psychological construction is another concern for religious scholars and psychologists.

## 1. Introduction

According to Qur’an (the holy book of the Islamic religion), fasting during Ramadan, the ninth month of Islamic Calendar, is one of the five pillars and is mandatory for all healthy adults and adolescent Muslims in Islam [1–4]. Ramadan fasting involves abstinence from food, drinking, oral medicine, smoking, and sexual activity from sunrise (Sahoor) to sunset (Iftar) [1–4]. The common practice is to take one meal in Sahur and Iftar [5]. The duration of fasting varies from 12 to 23 hours during the daytime for 29 or 30 days depending on different geographic regions and seasons [5].

There are exemptions from fasting according to the rules of fasting; the frail elderly, children before puberty, pregnant women or lactating or menstruating women, people with serious or chronic illness, or people traveling long-distance have the right to postpone fasting [3, 6, 7]. These individuals can continue fasting or fast during the next Ramadan based on their health condition. A large population of Muslims with diabetes insists on observing fasting despite exemptions and awareness of fasting risks [1, 8, 9]. According to an epidemiological survey conducted in 13 Muslim countries in 2004, 42.8% of people with type 1 diabetes (T1DM) and 78.7% with type 2 diabetes (T2DM) fasted for at least 15 days, and 67.6% of them fasted every day [10]. Approximately 50 million Muslims with diabetes worldwide fast very year [2]. In addition, approximately 1.6 billion (or 23%) of the world’s population are Muslims, and this number is growing at a rate of 3% per year [11]. Approximately 90 million Muslims are living with diabetes [3]. Studies reported that Ramadan fasting had no adverse effects on healthy people [12, 13], but it could lead to wide blood glucose fluctuations and increase the risks of acute metabolic complications, such as hypoglycemia, hyperglycemia, diabetic ketoacidosis, dehydration, and thrombosis, in the population with diabetes [2, 3, 14]. More seriously, two studies [3, 15] showed that it was more difficult and riskier for people with T1DM to manage their medical condition during Ramadan even under optimal Ramadan-specific education. This situation presents a great challenge and has captured the attention of health-care professionals (HCPs) [16].

In recent years, several review articles have been published regarding guidelines, management, and strategies for diabetes control during Ramadan fasting [2–4, 11, 16]. Most of them are based on small-sized quantitative studies and expert opinions. To meet the immediate needs for evidence-based practical guidelines for participants with diabetes during Ramadan fasting, the International Diabetes Federation (IDF) and the Diabetes and Ramadan (DAR) International Alliance organized a panel with renowned experts in this field to generate and deliver IDF-DAR Practical Guidelines for the management of diabetes during Ramadan [17]. The guidelines provide HCPs with practical information and skills to deliver medical care and support for their patients with diabetes during Ramadan fasting [17]. Although a great contribution has been made to review the extensive literature on the epidemiology and physiology of Ramadan fasting, risk stratification, structured education programs, modification of medications, types and application of anti-diabetic agents, methods of managing blood glucose level, and lifestyle adjustment for participants with diabetes during Ramadan to ensure that they could fast in a safer manner [2–4, 11, 16, 18], these reviews all obtained information or evidence from quantitative studies and experts’ opinion, whereas the subjective feelings of participants with diabetes on Ramadan fasting should also be emphasized. According to many studies, a number of participants with diabetes who observe Ramadan fasting never sought their general practitioners for advice or even exhibited reluctance to disclose fasting to HCPs [19–23]. To date, an increasing number of qualitative studies are targeting the patients’ experience, attitude, awareness, and perspective of issues relevant to Ramadan fasting. Evidence from these studies is of great importance in supplementing the current guidelines.

At present, systematic reviews have commonly been linked with quantitative meta-analyses, while reviews of data from qualitative research can also be performed and reported by applying a similar transparent, rigorous and reproducible methodology and presentation [24, 25]. Therefore, a meta-synthesis is appropriate when a systematic review intends to integrate qualitative studies [25]. The integration of multiple qualitative research results can explain the phenomenon more comprehensively, promote human-centered healthcare provision, and reflect the humanistic, social, and ethical characteristics of a phenomenon [26]. A meta-synthesis of qualitative research can combine data from different research backgrounds. Therefore, it can generate new theoretical or conceptual models to provide evidence for decision-making, implementation and evaluation of health-care interventions [26]. The research aim of our study is to review the experiences and views of people with diabetes on Ramadan fasting and to integrate valuable evidence for optimizing diabetes management from the perspective of qualitative research.

## 2. Methods

### 2.1 Research design

This study was a meta-synthesis of qualitative research where PubMed, Embase, Cochrane Library, Web of Science, Science Direct, CINAHL, PsycINFO, JBI (Joanna Briggs institute), SinoMed, CNKI, Wanfang and VIP network were searched, supplemented with manual searches of similar articles and references lists. Qualitative studies published in English and Chinese language were included from inception to December 30, 2019. Studies were eligible when they evaluated experience and perceptions on Ramadan fasting for people with diabetes. The quality appraisal of potential studies selected for selection criteria was assessed using the Critical Appraisal Skills Program (CASP) tool [27]. The strategy of inductive thematic analysis was used to identify key themes and categories from the included studies. The themes and extracted information from the included studies were analyzed to form new descriptive or analytical point of views, and then the experiences and views between the studies were synthesized as new explanation and description [26]. Our meta-synthesis methodology was framed and informed by the Enhancing Transparency in Reporting the Synthesis of Qualitative Research (ENTREQ) statement [28, 29]. The confidence of the integrated new themes was assessed to place in findings from the qualitative synthesis.

### 2.2 Inclusion and exclusion criteria

SPIDER (Sample, Phenomenon of Interest, Design, Evaluation, Research type) tool was inspired from the PICOS model of quantitative study and designed for constructing qualitative research questions by Cooke A, et al. [30]. We selected studies based on our aims and used SPIDER tool [31] to formulate inclusion criteria, and the exclusion criteria was made through pilot literature (Table 1).

**Table 1:** Inclusion and exclusion criteria of literature.

Inclusion criteria (SPIDER) [30, 31]	Exclusion criteria
**• S**: people with diabetes (only T1DM and T2DM) experiencing Ramadan fasting **• PI**: experience (or perspective, attitude, knowledge, perception, etc.) of religious beliefs, medication, health education, health care professionals (HCP), etc. related to Ramadan fasting **• D**: interview, focus group, group discussion, questionnaire **• E**: thematic analysis, content analysis, ethnography, narrative, descriptive, etc. **• R**: qualitative research or mixed methods	**•** Irrelevant topics **•** Pure quantitative study **•** No review or protocol or animal study **•** Non published journal articles **•** Non-English or non-Chinese **•** Unavailable full-text

S = Sample, PI = Phenomenon of Interest, D = Design, E = Evaluation, R = Research type

### 2.3 Search strategy

Based on the SPIDER model (Table 1), the following keywords were combined for the retrieval, and the Chinese keywords were similar to the English keywords as follows:

(diabetes OR diabet* OR Diabetes Mellitus OR diabetic) AND (fast* OR Ramadan OR Ramazan OR Islam* OR Muslim*) AND (qualitative OR narration OR narrative OR narrati* OR descriptive OR description OR descripti* OR thematic OR thematic OR thematically OR thematic* OR grounded theory OR content analysis OR content analy* OR ethnography OR ethnograph* OR interview OR interview* OR focus group OR phenomenon OR phenomenol*)

For example, the PubMed search strategy was performed as follows:

#1 (title) fast* OR Ramadan OR Ramazan OR Islam* OR Muslim*

#2 (title) diabet*

#3 (Mesh term) "Diabetes Mellitus"

#4 (title/abstract) qualitative OR narrati* OR descripti* OR interview* OR thematic* OR “grounded theory” OR “content analy*”

#5 (Mesh term) "Qualitative Research"

#6 #2 OR #3

#7 #4 OR #5

#8 #1 AND #6 AND #7

Two authors (JY Liao & HT Xie) independently and simultaneously searched 12 electronic databases (PubMed, Embase, Cochrane Library, Web of Science, Science Direct, CINAHL, PsycINFO, JBI, SinoMed, CNKI, Wanfang, and VIP). After each database was retrieved, the data were immediately discussed to gain timely information. If a discrepancy was encountered, the third reviewer (Z Li) would make the final decision. The twelve databases were searched from inception to December 30, 2019. Additionally, similar articles and references of the retrieved articles were searched manually to identify other potential articles.

### 2.4 Study selection

Two authors (JY Liao & HT Xie) independently used NoteExpress V3.0 software to import retrieved articles, remove duplicates, and conduct title and abstract screening; thereafter, full-text articles were downloaded for rescreening to meet the inclusion and exclusion criteria. During this process, any disagreement was discussed or resolved by the third reviewer (Z Li).

### 2.5 Study quality appraisal and data extraction

#### 2.5.1 Quality appraisal

Strict quality evaluation is required for the final included articles. The evaluation content of qualitative studies for meta-synthesis includes research aim, methods, and design (research objects, sampling methods, recruitment strategy, data collection and data analysis) [32]. In addition, researchers need to evaluate the risk bias, identify the source of risk bias, and analyze the impact of the risk bias on the results of systematic reviews [33]. We chose CASP (Critical Appraisal Skills Program) [27] as the risk bias tool for rigorous evaluation based on our research design and characteristics.

Each article was read and appraised independently by two authors (JY Liao & HT Xie) using CASP to examine the study validity, adequacy and potential applicability of the results. We classified the study quality into three levels, including high quality, moderate quality and low quality. A high-quality study met all the requirements of the ten items in the CASP, and a low-quality study indicates that at least two items of the ten items were not met, especially inappropriate research design. A study with a moderate level of quality indicates that the studies were not classified as high or the low quality. Only studies of high and moderate quality were included.

#### 2.5.2 Data extraction

While evaluating the study quality, data extraction was simultaneously conducted. The following information was extracted: name of the first author, publication year, country, author discipline, study design, study aim(s) or issue(s), study setting, recruitment strategy, sample characteristics, data collection methods, data analysis strategy, ethical issues, methods of trustworthiness, data saturation, relationship between researcher and participants, and statement of findings.

### 2.6 Data analysis and synthesis

To perform data analysis and synthesis, a thematic analysis was conducted. All the authors used an iterative process to read and comprehend the results of all the primary articles. Next, the two authors (Z Li & JY Liao) interpreted the results and marked the same or similar findings to form new themes using an inductive thematic approach. Then, raw data, original quotations and analysis were assessed to modify the newly formed themes to verify that they were accurate and adequate. Finally, new themes were combined and concluded to identify more broad concepts of new categories, which could reflect the phenomenon more comprehensively and deeply [26]. Each procedure was completed through discussion and verification among all the authors.

Audit trail process: We printed five copies of the 11 included articles for the five authors. Each author read the article dependently. Z Li was mainly responsible for labeling five articles in green color, and JY Liao was mainly responsible for labeling the other six articles in red color independently. Then, Z Li and JY Liao exchanged labeling the two groups of articles on the basis of the former labeling. After labeling twice, each article was marked in green and red colors. Then, JY Liao wrote down all the labeled original themes and quotes, which were assessed by HT Xie in red color. Based on the excerpts, all the authors discussed generating new themes and categories.

### 2.7 Confidence grading of the integrated new themes

The Confidence of the Evidence from Reviews of Qualitative Research (CERQual) approach is targeted at evidence grading of qualitative systematic reviews and is based on four aspects of the assessment: methodological limitations, relevance, coherence and adequacy of data [34]. It grades the confidence of individual review findings as high, moderate, low or very low confidence [34]. We used it to grade the confidence of the new themes. For methodological limitations, we took results from quality appraisal with CASP. With regard to relevance, we identified the correlation of original study aims and study objects with our research objectives. For coherence, we checked the similarities between the included studies. For the adequacy of the data, we inspected the original full text.

## 3. Results

### 3.1 Results of literature retrieval

**Fig 1:**
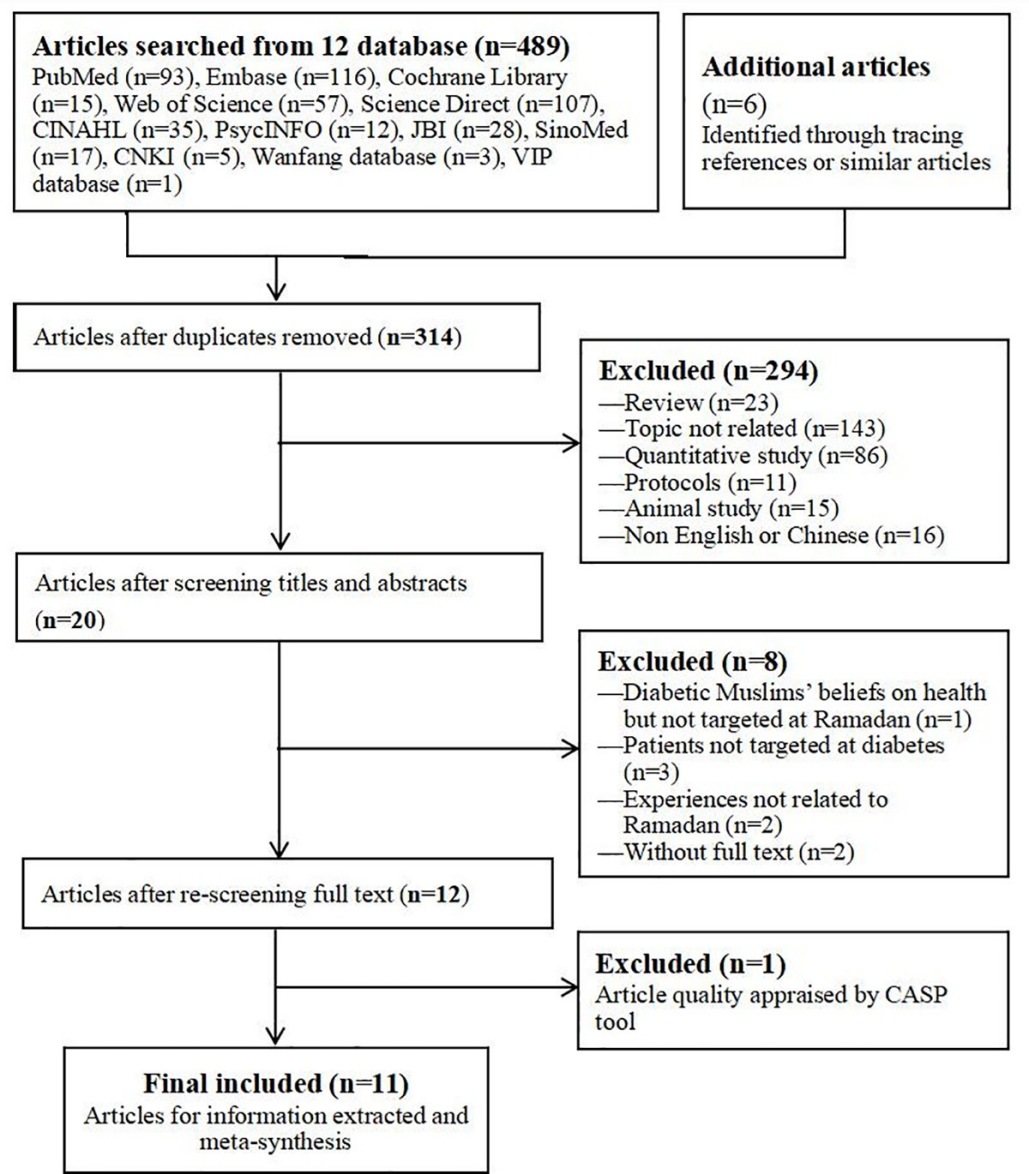
Flow chart of the literature search and screening.

The study selection process is shown in Fig 1. At first, a total of 489 articles and another six articles were searched from the initial retrieval, among which all the studies in the Chinese databases (SinoMed, CNKI, WanFang and VIP) were excluded. Thus, it could be inferred that no such research was conducted in China although there are Muslims who also fast during Ramadan [35]. Next, after removing duplicates and excluding articles at odds with the inclusion and exclusion criteria, 20 articles remained. Then, eight articles were excluded after screening the full text for various reasons. After screening the full texts of the 12 articles and performing the quality appraisal, 11 articles were finally included for subsequent meta-synthesis.

### 3.2 Quality appraisal results

A final integrated appraisal table was made through subsequent discussion (Table 2) by the two authors (JY Liao & HT Xie). As a result, only one article was excluded due to an unclear recruitment strategy or data collection. Apart from that, participants in this study were not specific to participants with diabetes, and the results were mixed together.

**Table 2:** Study quality appraisal details by the CASP tool.

1^st^ author (year)	①	②	③	④	⑤	⑥	⑦	⑧	⑨	⑩	Overall appraisal	Result
El-Rahman 2019 [36]	1	1	1	1	1	1	1	1	1	1	high quality	included
Alsaeed 2019 [19]	1	1	1	1	1	1	1	1	1	1	high quality	included
Alluqmani 2019 [37]	1	1	1	1	X	1	1	1	1	1	high quality	included
Alsaeed 2019* [38]	1	1	1	1	1	1	1	1	1	1	high quality	included
Patel 2015 [20]	1	1	1	1	1	1	1	1	1	1	high quality	included
Al Slail 2018 [21]	1	1	1	1	1	0	1	1	1	1	moderate quality	included
Peterson 2012 [22]	1	1	1	1	1	0	1	1	1	1	moderate quality	included
Mygind 2013 [39]	1	1	1	1	1	0	X	1	1	1	moderate quality	included
Myers 2019 [40]	1	1	1	1	1	1	1	1	1	1	high quality	included
Almansour 2018 [23]	1	1	1	1	1	1	1	1	1	1	high quality	included
Darko 2019 [42]	1	1	1	X	X	0	1	1	1	1	low quality	excluded
Lee 2017 [43]	1	1	1	1	1	1	1	1	1	1	high quality	included

① Was there a clear statement of the aims of the research? ② Is a qualitative methodology appropriate? ③ Was the research design appropriate to address the aims of the research? ④ Was the recruitment strategy appropriate to the aims of the research? ⑤ Was the data collected in a way that addressed the research issue? ⑥ Has the relationship between researcher and participants been adequately considered? ⑦ Have ethical issues been taken into consideration? ⑧ Was the data analysis sufficiently rigorous? ⑨ Is there a clear statement of findings? ⑩ How valuable is the research?

*This study shares the same authors with the second study but different in content.

1 indicates “Yes”; 0 indicates “No”; X indicates “Can’t tell”.

### 3.3 Characteristics of the included articles

A final integrated data extraction table was made through subsequent discussion (see Table 3). Of the 11 included studies, all were published within the recent decade (from 2012 to 2019). Five articles were published in 2019, which indicated that the included articles were new and that the topic is a front-burner issue. The studies were conducted in eight countries, including Kuwait, Saudi Arabia, Australia, Malaysia, Egypt, the UK, Denmark and the USA. For the study design, almost all studies were based on qualitative interviews. Only one study [37] applied mixed-method approaches, and accordingly, only the qualitative components were included in the meta-synthesis [44]. For the aims, the main concerns were participants’ subjective feelings and some phenomena. The main recruitment strategies of the studies were purposive sampling and convenience sampling.

**Table 3:** Data extraction of the 11 included articles.

Author, publication year	Country, author discipline (number), study design	Study aim(s)/issue(s)	Study setting, recruitment strategy	Sample characteristics (diabetic type (disease duration), sample size (male, female), age, medication, fasting days)	Data collection method, data analysis strategy, ethical issues	Method of trustworthiness, data saturation, relationship between researcher and participants, statement of findings
El-Rahman et al., 2019 [36]	**•** Egypt **•** Nursing (3) **•** Descriptive qualitative study	**•** To explore the experiences and needs of adults with type 2 diabetes during Ramadan fasting. **•** Research questions: 1) What are the experiences of people living with diabetes during Ramadan fasting? 2) What are the needs and desires of these individuals with respect to Ramadan fasting?	**•** In the meeting room of the diabetes outpatient clinic in Port Said, a familiar and relaxed atmosphere **•** Purposive sampling	• T2DM (at least 6 months) • 30 participants (10 males, 20 females) • Mean age = 50.8yrs (30~62yrs); • Oral hypoglycemic agents: sulfonylureas • Intended to fast for at least 15 days	• Semi-structured interviews (individual in-depth interviews) • Thematic analysis • Verbal consent, informed consent, approved by the Institutional Review Board	• Credibility & dependability & confirmability • data saturation No reported • Clear statement of findings
Alsaeed et al., 2019 [19]	**•** Kuwait **•** Pharmacy (1), sciences and Technology (1), medicine (1) **•** Qualitative study	**•** To explore how the Dose Adjustment for Normal Eating (DAFNE) course affected people with type 1 diabetes’ fasting experiences to provide insight into the benefits of structured education for people wishing to fast while managing their diabetes	**•** Interviewed at DDI, which is where the DAFNE courses take place **•** Purposive sample	• T1DM (average duration = 17.8yrs) • 40 participants (No reported) • Mean age = 33yrs (21~53yrs) • With 23 Multiple daily injections (MDI) users and 17 insulin pumps • Average total fasted days is 25.6 days	• Semi-structured interviews • analyzed thematically • Ethical approval from the Ministry of Health	• No reported • Data saturation • Relationship stated • Clear statement of findings
Alluqmani et al., 2019 [37]	**•** Saudi Arabia **•** Pharmacy (14) **•** Mixed-method study	**•** To identify any drug-related problems (DRPs) in diabetic participants during Ramadan fasting in Saudi Arabia	**•** Can’t tell **•** Convenience sample	• T2DM (inferred from medication) • 20 participants (10 male, 10 female) • No reported • No reported • No reported	• Face-to-face semi-structured interviews • Thematic analysis • Approved by Research Ethics Committee and by Hospital Diabetic Center & informed consent	• No reported • No reported • No reported • Clear statement of findings
Alsaeed et al., 2019* [38]	**•** Kuwait **•** Pharmacy (1), sciences and Technology (1), medicine (1) **•** Qualitative approach	**•** To pilot a Ramadan‐focused educational module based on DAFNE principles to educate graduates about safe fasting practices; **•** To gain insight into the challenges associated with fasting and to provide advice on how to ameliorate such challenges	**•** At DDI three days before the start of Ramadan, in a closed room **•** Can’t tell	• T1DM (No reported) • 47 participants (No reported) • No reported • Insulin pumps and MDI • No reported	• Group discussion • Thematic analysis • Ethical approval from the MOH & informed consent	• No reported • Data saturation • Relationship stated • Clear statement of findings
Patel et al., 2015 [20]	**•** UK **•** Psychology (1), public health (4), medicine (1) **•** Qualitative study	**•** To explore beliefs and experiences of fasting during Ramadan, **•** To explore perceptions of Muslim people with diabetes the role played by their general practitioner (GP) and/or practice nurse (PN) in supporting them to fast.	**•** General practices and community groups located in Greater Manchester **•** Random and purposive sampling techniques	• T1DM (2) and T2DM (11) (No reported) • 13 participants (No reported) • Mean age = 52yrs • T1DM: 2 insulin injection; T2DM: 11 oral medication • No reported	• Semi-structured interviews • Thematic analysis using a constant comparison approach, grounded theory • Ethics approval from National Research Ethics Service and a Research Ethics committee	• No reported • Data saturation • No reported • Clear statement of findings
Al Slail et al., 2018 [21]	**•** Saudi Arabia **•** Public health (4) **•** Qualitative study	**•** To explore the health status of people with diabetes during Ramadan, **•** To explore issues that adversely affect the health of people with diabetes during Ramadan	**•** Casablanca Health Center in Riyadh, Saudi Arabia **•** Purposive sampling techniques	• T1DM and T2DM (18yrs +; Insulin-dependent >5yrs) • 15 participants (8 males, 7 females), • (18~73 yrs.) • T1DM: insulin; T2DM: oral antihyperglycemic drugs, insulin, or both • All participants fasted the last month of Ramadan.	• Focus group discussions • Thematic analysis • Informed consent & ethics approval from Institutional Review Board	• No reported • No reported • No reported • Clear statement of findings
Peterson et al., 2012 [22]	**•** Australia **•** nursing and midwifery (3) **•** Phenomenological study, phenomenology	**•** In this case an understanding of being a Muslim person with diabetes during Ramadan will assist health professionals to work with clients incorporating their beliefs and practices while promoting optimal management.	**•** Two held at the Mosque and two in participants’ homes **•** Poster advertising	• T2DM • 4 participants (3 males, 1 female), • mid-forties to early 60’s, • No reported • No reported	• Unstructured interview • Can’t tell • Written informed consent & ethics approval from institutional Review Board	• No reported • Data saturation • No reported • Clear statement of findings
Mygind et al., 2013 [39]	**•** Denmark **•** Pharmacy (2), public health (2) **•** Interview study	**•** To explore patient perspectives on medicine use during Ramadan, reasons for fasting and experiences with counselling on medicine use during Ramadan among people of Pakistani background with type 2 diabetes and at least one other chronic condition.	**•** Greater Copenhagen **•** Purposive sampling	• T2DM (3~20yrs) • 6 participants (1 male, 5 females) • age 42~69 • anti-diabetics: metformin, insulin and/or sitagliptin • No reported	• Semi-structured interviews and medication reviews, and field notes • Hermeneutic approach, thematic analysis (the authors did not say directly) • Consent form & approved by a Data Protection Agency	• Validity • No reported • No reported • Clear statement of findings
Myers et al., 2019 [40]	**•** United States **•** Nursing (4) **•** Qualitative study	**•** To explore the beliefs which influence the experience and practices of diabetes management among Muslims in the United States during Ramadan, **•** To explore perspectives of Muslims with diabetes on their experience with health-care providers providing support during their fasting experience.	**•** In a private room provided at the mosque they attended **•** Purposive sample	• T2DM (No reported) • 14 participants (9 males, 15 females) • Mean age = 51yrs, 43~75yrs • No reported • No reported	• Semi-structured interview • Constant comparative analysis, thematic analysis • Approved by an Institutional Review Board.	• Credibility & dependability • Data saturation • Relationship stated • Clear statement of findings
Almansour et al., 2018 [23]	**•** Australia **•** Pharmacy (3) **•** Qualitative, exploratory design	**•** To understand their experiences, health-related needs and service preferences regarding diabetes management	• No mentioned • Purposive convenient sample	• T2DM (No reported) • 25 participants (No reported) • No reported • No reported • No reported	• Semi-structured interviews • Thematically analyzed using the grounded theory approach • Ethics approval from a Research Ethics Committee.	• No reported • Data saturation • No reported • Clear statement of findings
Lee et al., 2017 [43]	**•** Malaysia **•** Pharmacy (2), pediatrics (1), public health (2) **•** Qualitative study	**•** To explore the beliefs, experience and diabetes management of Muslim people with diabetes who choose to fast during Ramadan in Malaysia	**•** Conducted at a time and location that was convenient for participants **•** Purposefully sampled	• T2DM (at least 6 months, HbA1c between 7.5% and 11%) • 53 participants (No reported) • Aged 18–75yrs • No reported • Fasting at least 15 days during Ramadan	• Semi-structured focus group interview • Thematic approach • Approved by a Research Ethics Committee & informed consent	• No reported • Data saturation • No reported • Clear statement of findings

For the sample characteristics of the studies, seven studies included only T2DM participants (152 participants) [22, 23, 36, 37, 39, 40, 43], two studies included only T1DM (87 participants) [19, 38], and the other two studies included both T1DM and T2DM (28 participants) [20, 21]. Regarding medication, for T2DM participants, anti-diabetics, such as metformin, sulfonylureas and sitagliptin, insulin, or both oral medication and insulin, were reported. For T1DM participants, multiple daily injections (MDIs) and insulin pumps were reported. For fasting days, four studies [19, 21, 36, 43] reported that, the average duration was at least 15 days, and participants in one study fasted the entire month [21]. For data collection and analysis, eight studies used the semi-structured interview method [19–21, 23, 36–47], two applied group discussion or focus group discussion [21, 38], and one used unstructured interview [22]. For the data analysis strategy, two studies [22, 39] did not report this information, whereas the others all applied thematic approaches. Almost all studies were approved by a Research Ethics Committee or an Institutional Review Board. In addition, verbal consent and written informed consent were also obtained in some studies. With respect to the methods of trustworthiness, only three studies [36, 39, 40] mentioned credibility, dependability, confirmability and validity. Data saturation was discussed in eight studies [19, 20, 22–23, 36, 38, 40, 43]. Regarding the relationship between researchers and participants, only three studies critically examined the authors’ role [19, 38, 40]. All the studies provided a clear statement of the findings.

### 3.4 Meta-synthesis

**Fig 2:**
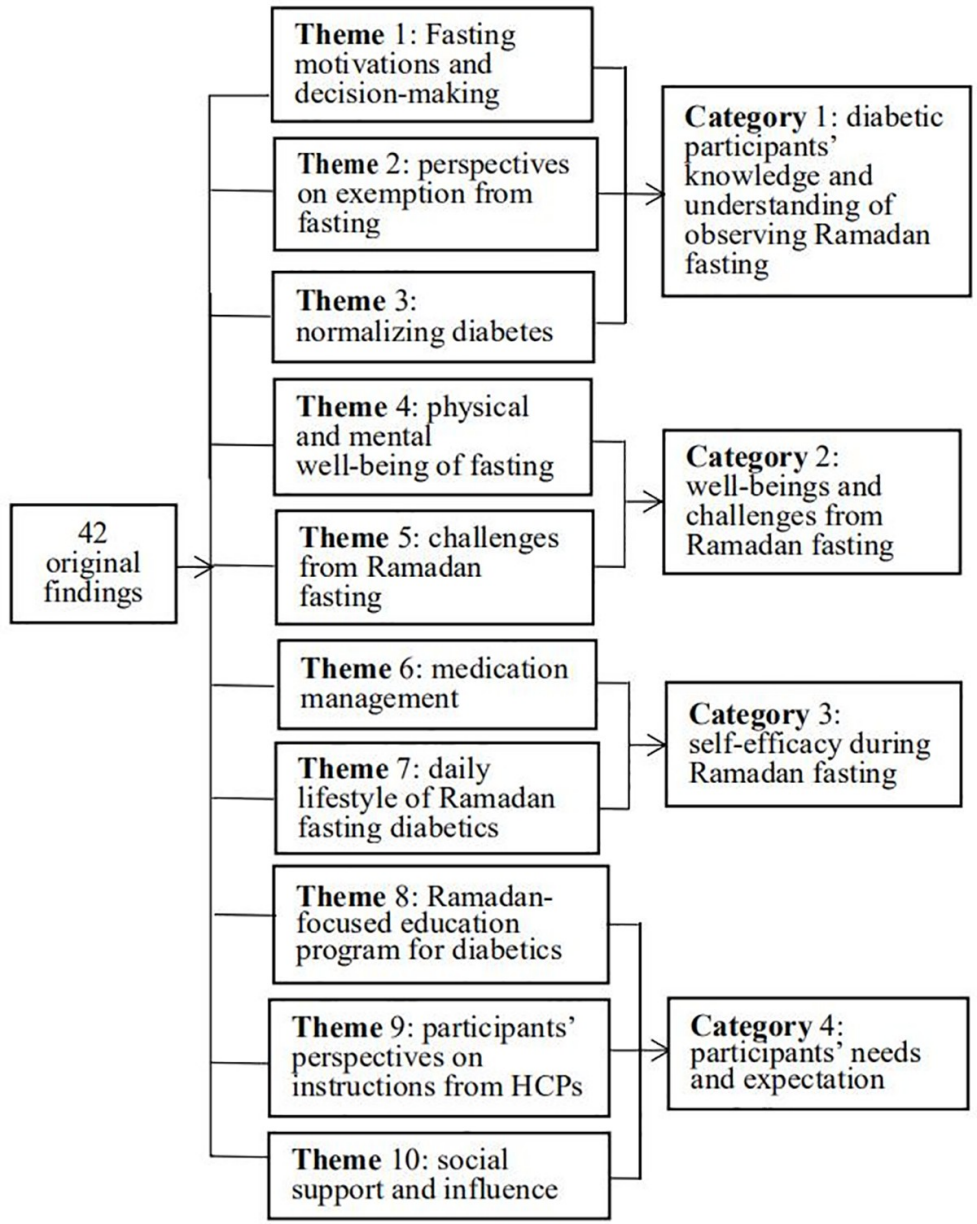
New themes and categories from 11 included articles.

Based on a meta-synthesis of the findings of the 11 included studies, 43 findings were extracted (Table 4), and similar results were summarized and combined to form ten new themes. The ten new themes were integrated into four categories (Fig 2).

**Table 4:** Extraction of original findings and integrated new themes and categories.

Author (year)	Findings (themes/categories)
El-Rahman et al., 2019 [36]	(1) Theme 1: Participants’ experience during fasting months • Optimum sense of physical well-being, (2) Theme 2: Behavioral and coping strategies during fasting • Restriction in daily activities • Dietary modification • Self-management and self-efficacy • Medication modification (3) Theme 3: health-care support and needs
Alsaeed et al., 2019 [19]	(1) Reduction in fluctuations and complications, (2) Improvement in confidence and self-reliance, (3) Tailored support for dose and pump programming adjustments, (4) Positive effect on well-being, (5) Encouraging informed-decision making about fasting
Alluqmani et al., 2019 [37]	(1) Patient-Related Factors • Management of Diabetes during Ramadan • Diet during Ramadan • Lifestyle during Ramadan • health-care Professionals-Related Factors • Communication between health-care Professionals and Patients • Future considerations for fasting • Seeking advice before making changes in medicine schedule
Alsaeed et al., 2019* [38]	(1) Motivations for attending the workshop, (2) Past experiences with fasting, • Fasting pre‐DAFNE education • Social pressure and stigma • Ramadan conditions • Positive experiences • Strategies and practices adopted (3) Impact of DAFNE on fasting experience, (4) Expectations regarding fasting during the year of the study
Patel et al., 2015 [20]	(1) normalizing diabetes, (2) the significance of fasting, (3) pressure to fast and not fast, (4) and to disclose or not to disclose
Al Slail et al., 2018 [21]	(1) Knowledge, attitudes, and practices (2) Health seeking behaviors (3) Assessment of diet (4) Physical activity (5) Capacity (regarding satisfaction with quality of health-care service)
Peterson et al., 2012 [22]	(1) Knowing and understanding: being in harmony with the body, • including understanding its capabilities, limitations and responses to change. (2) Control: being in charge of diabetes when fasting during Ramadan (3) Acceptance and recognition: the relationship between diabetes and fasting during Ramadan (4) Faith and belief: the use of courage and conviction
Mygind et al., 2013 [39]	Five themes emerged from the analysis: (1) exemption from fasting; (2) medicine use during Ramadan; (3) feeling of improved well-being when fasting; (4) explanations for improved well-being; • physiological aspects • social aspects • religious aspects (5) talking with others about fasting.
Myers et al., 2019 [40]	Two main themes: (1) having diabetes and fasting during Ramadan, comprising four ‘‘feelings”: • feeling spiritually connected, • feeling socially connected, • feeling physically healthy, • feeling religiously obligated. (2) fasting challenges, including: • feeling sick and dehydrated, • feeling vulnerable and poorly understood by health-care providers.
Almansour et al., 2018 [23]	(1) Religion versus health–a dichotomy of values and practices, (2) Impact of fasting, (3) Self-efficacy (4) Sociocultural norms versus healthy lifestyles, (5) Patients perspectives about HCPs, • Preference for advice from co-religious health professionals, • Accessibility, • lack of trust and awareness of pharmacists’ role, (6) Education and awareness needs • Information needs
Lee et al., 2017 [43]	(1) perception of Ramadan, (2) managing side effects during Ramadan, (3) diet control during Ramadan
Total	43

**Category 1.** Knowledge and understanding of observing Ramadan fasting

**Theme 1.** Fasting motivations and decision-making

From analyzing the quotations of the participants in the 11 studies, most participants took the initiative to celebrate fasting, and only a small portion were reluctant due to social pressure [20]. Their motivations could be summed up in three aspects: performing religious obligation [20, 22], grasping the opportunity to regulate body and mental state [36, 38, 39], and learning from and communicating with other participants about fasting techniques [38]. The participants explained that the Qur’an provided guidance to make the decision to fast [22]. There were three groups of participants. Group 1: Despite their disease condition, these participants insisted on fasting [20, 21]. Group 2: These individuals combined their own understanding of diabetes to determine whether to fast [22]. Group 3: These participants took a wait-and-see approach and tried fasting as long as possible [36].

**Theme 2.** Perspectives on exemption from fasting

Some inconsistencies were noted when referring to whether fasting was obligatory. In several studies, some participants responded in a relaxed way that people with diabetes could be exempted from fasting [39]. However, a minority of participants replied that fasting was compulsory even for pregnant females [21]. Two participants said that Islam was not so strict that people with T2DM could temporarily postpone fasting [39]. The other two participants indicated they had no idea [21].

**Theme 3.** Normalizing diabetes

Regarding attitudes towards diabetes, many participants took their disease for granted. Some participants did not perceive themselves as being ill [36]. Some participants minimized the seriousness of the disease and ignored the importance of self-management [20]. In a study conducted in Australia, greater than half of the participants underestimated their health in comparison with committing religious obligations [23]. In a study of interviews with South Asian participants with diabetes, the participants held a strong belief that diabetes was a normal part of their lives [20].

**Category 2.** Well-being and challenges from Ramadan fasting

**Theme 4.** Physical and mental well-being

Although some participants with diabetes abstained from eating and even discontinued their medication, they gained both positive physical and mental well-being. The reasons could be explained by physiological, religious and social aspects [39]. Regarding the physical benefits, many participants indicated that fasting benefits their overall health, i.e., better diabetes management, control of blood glucose levels, body weight loss, and lowering levels of cholesterol [36, 39, 40, 43]. Some participants reported waited for the Ramadan month like celebrating a festival or observing a holiday. Mental benefits were mainly obtained from their strong faith in their God Allah. Some participants felt confident because they thought Allah gave them power and strength to pull through those days [36, 39]. The participants tolerated the fasting very well and felt happy and energetic [36]. They felt spiritually connected to Allah and felt a connection with suffering people to achieve a type of spiritual sublimation. In addition, they also felt socially connected. For example, participants had an opportunity to reunite with family [43].

**Theme 5.** Challenges from Ramadan fasting

Difficulties, side effects, complications, risks or any other unexpected incidents related to Ramadan fasting could be classified into challenges. Challenges were noted in all 11 studies and were categorized as follows (a) feeling hungry, sick, dehydrated, lethargic and vulnerable, (b) experiencing blood glucose fluctuations, or hospital admission for diabetic ketoacidosis, (c) anxiety or fears of their body condition, (d) poorly understood by HCPs and lacking support from family or friends, (e) unwilling to disclose to non-Muslims and (f) symptoms, such as gastrointestinal tract disturbances, headache, and sleep disorders. The aforementioned challenges were not mutually exclusive. For example, anxiety or fears of hypoglycemia lead to extreme hunger, fatigue, trembling, sweating, or binge eating [21, 23, 43].

**Category 3.** Self-efficacy during Ramadan fasting

According to the report from almost all of the included studies [19, 21, 23, 36–39, 43], we defined self-efficacy as a participant’s belief or skills to successfully manage medication adjustment, regular blood glucose monitoring, diet modification, changes in physical activity and other relevant lifestyles. Self-efficacy could be presented in two respects: medication management and daily lifestyle.

**Theme 6.** Medication management

Regarding the medication time, the majority of participants reported that they administered hypoglycemic drugs or insulin before meals, namely, before Sahoor (the last meal before fasting starts) or before Iftar (breaking fasting at sunset to have meal) [21]. Some explained that they took medicine daily depending on their circumstances, i.e., their blood glucose level or free time [36]. Regarding medication doses, the majority of participants were self-reliant. They adjusted the doses or even left out medication [19, 23, 36, 38]. Only a few followed their doctors’ instructions to change the doses [21, 23].

**Theme 7.** Daily lifestyle

Diet modification is of great concern since dietary mode has a direct impact on blood glucose. Therefore, food types and intake were routinely discussed. Participants’ diets during Ramadan are different from meals at ordinary times. The diet during this occasion has special features and is often rich in carbohydrates and fat [21, 36]. In two studies, all participants reported that they broke fasting to have meal with dates. However, participants held contradictory views on this practice. One side believed that dates could cause poor glucose control [36], whereas the other side stated that sweet dates could help restore normal blood glucose levels [21]. Regarding food intake, binge eating was described in some participants who could not withstand food temptation, especially food rich in sugar and fat [21, 23, 43].

Another lifestyle change was physical activity. Conflicting opinions were reported. Participants who feared hypoglycemia supported a decrease in daily activity to preserve energy [36, 38]. However, some participants who supported increased movements were physically active, and they thought that sports could offer benefits by reducing blood glucose [37]. Participants with a neutral point of view neither approved heavy exercise nor objected to daily activity, and they regarded walking as a good choice during Ramadan [37].

**Category 4.** Participants’ needs and expectations

**Theme 8.** Ramadan-focused education program

In the 11 studies, a group of three authors contributed two different articles targeted at T1DM participants with an education program named DAFNE (Dose Adjustment for Normal Eating), which taught participants skills, such as how to alter medication doses, count carbohydrates, and adjust fast-acting insulin dose based on their calculation [19, 38]. Both studies were performed after DAFNE. One was performed after Ramadan [19], whereas the other was performed before Ramadan [38].

Up to 95% of participants reported that Ramadan-focused education was important [38]. A number of participants urged the introduction of an education program [23, 38]. Participants who graduated from the DAFNE course gave positive feedback [19, 38]. The majority did not seek medical information after attending DAFNE because they could fast in a safe manner. However, 9.4% participants said instruction from HCPs was enough, and extra education was redundant [43].

**Theme 9.** Participants’ perspectives on instructions from HCPs

In several studies, the role of HCPs was controversial to many participants. Some participants would like to seek advice from HCPs on condition that the HCPs were trained, empathetic and realized religious significance [20, 36]. Several participants turned to HCPs but with the response of not recommending fasting or leaving them to make their own decision [19, 36]. A few participants reported that they could receive valuable information from HCPs [43]. Many Muslims refused to consult HCPs for the following reasons: (a) fear of being advised not to fast [20], (b) being confident and self-reliant due to previous experiences [20], (c) thinking HCPs were incapable of guiding them due to a lack of expertise and deficient awareness of Muslim faith [20, 21, 37]. Participants were even unwilling to disclose fasting to HCPs, especially pharmacists, and dissatisfied with their roles [19, 20, 23].

**Theme 10.** Social support and influence

In some studies, participants reported experiencing social pressure, especially from their family members and work colleagues [20]. Some noted that although they were exempted from fasting, they felt guilty and uncomfortable eating before their fasting family members [20]. Some pointed out the tensions between their decision not to fast and the pressure received from their family to fast [20]. However, some family members persuaded them to quit fasting considering the potential adverse events. Similarly, participants who chose to conceal their diabetes or fasting behavior at the workplace were at risk of causing health problems, such as hypoglycemia. It was evident that tensions could be alleviated with support from society during this religious celebration [20, 23, 38].

### 3.5 Confidence grading of the integrated new themes

Each of the ten themes was graded based on four aspects: methodological limitations, reference, coherence and adequacy of data. The overall evidence grading was conducted based on the four mentioned aspects. The final evidence of each new theme is presented in Table 5.

**Table 5:** Evidence grading of the integrated 10 themes with CERQual.

Themes	Study number [study source]	Methodological limitations	Relevance	Coherence	Adequacy of data	Overall evidence grading
Theme 1	Seven [20–22, 36, 38–40]	moderate	high	high	high	high
Theme 2	Two [21, 39]	moderate	high	moderate	low	moderate
Theme 3	Three [20, 23, 36]	high	high	high	high	high
Theme 4	Four [36, 39, 40, 43]	high	high	high	high	high
Theme 5	Seven [20–23, 36, 38, 39, 43]	high	high	moderate	low	moderate
Theme 6	Four [19, 21, 23, 36]	high	high	high	high	high
Theme 7	Five [21, 36–38, 43]	high	high	moderate	moderate	moderate
Theme 8	Four [19, 23, 38, 43]	high	high	unclear	low	moderate
Theme 9	Seven [19–21, 23, 36, 37, 43]	high	high	moderate	high	high
Theme 10	Three [20, 23, 38]	high	high	high	moderate	high

## 4. Discussion

### Relationship between T1DM and T2DM in Ramadan fasting

Based on the aim of our study, we included and combined both T1DM and T2DM participants because they experience the same Ramadan fasting atmosphere and share many common experiences and views on Ramadan fasting itself. According to the reported details of participants’ medication, participants with T1DM all administered insulin (insulin pump or MDI), while participants with T2DM administered oral hypoglycemic agents, insulin, or both. Some T2DM participants may exhibit a longer course of diabetes, and they may gradually lose islet function. More severe islet B cell failure is associated with reduced effectiveness of oral hypoglycemic agents. Thus, insulin should also be used for treatment. Regarding insulin-dependence, the exemption and reduction of medication for insulin or oral hypoglycemic drugs in TIDM and T2DM are overlapped to some extent. A previous study demonstrated that living with diabetes did not inhibit people from fasting, especially those with T2DM treated with oral medications [20]. Few people on insulin observed fasting compared with those on oral medications [10]. Thus, the administration of insulin is a factor that influences the decision to fast for people with diabetes. Interestingly, studies [19, 38] demonstrated that if T1DM individuals received Ramadan-focused education, they were more willing to fast because they were more capable of managing their diabetes. This finding also reflects that people with diabetes on insulin need more professional instructions than those on oral medication. Participants with T1DM credited their glycemic control and fewer complications to tailored educations [19, 38]. A study [41] also showed that structured education was associated with a 61% decrease in the ketoacidosis risk in adults with T1DM. Therefore, Ramadan-focused education for both T1DM and T2DM participants, especially those of insulin-dependent individuals, is an urgent need.

### Doctor-patient and religion relations (health and religion)

Ramadan fasting is an activity with both religious and social meaning [19, 23]. It is dubious whether or not patients trust their doctors and whether they attach more importance to their religious God or their physical health. Generally, as shown in category 2, although Ramadan fasting is challenging, its well-being is evident, which concurs with the results from quantitative studies indicating improvements in glycemic control, sleep duration and physical activity [45, 46]. Themes in the other three categories deserve special attention. For category 1 (themes 1, 2, and 3), some participants persistently observed fasting due to their devout faith regardless of their physical health and fasting exemption rules. Their incorrect perceptions expose them to numerous health risks. For example, as shown in category 3 (themes 6 and 7), frequent blood glucose monitoring was of great importance [2, 17], but it was neglected or ignored by some participants for reasons of either being too busy or thinking that pricking their fingers indicated breaking fasting [2]. Some participants with T1DM regard insulin injection as nullification of fasting [21]. Another aspect is diet regulation and control. In general, rich foods are provided at the beginning and end of the fast to maintain the fasting duration [5]. Participants with diabetes should drink sufficient fluids and avoid foods rich in sugar and fat [47]. However, some participants lose control and binge eating, which might lead to hyperglycemia. Timely and correct instructions are not accessible to all fasting diabetic Muslims. Non-Muslim HCPs may not advise patients to fast. Religious leaders may not clarify that routines, such as pricking fingers and insulin injection, do not nullify fasting. A large communication gap exists among patients, doctors, and religious leaders, especially the doctor-patient relationship, including the doctor’s duty to care and the patient’s autonomous decision [48]. Some HCPs made their patients decide whether to fast. Many participants underestimated their doctors’ roles. This situation was reported repeatedly and was attributed the following reasons: (a) many non-Muslim doctors were unaware of Islamic culture, and they discouraged fasting to lower health risks for their patients; (b) many participants showed a gap in their knowledge regarding the responses to medication adjustment; (c) some participants distrusted HCPs’ expertise, and they were particularly dissatisfied with pharmacists. The relationship between Muslims with diabetes and HCPs is actually a reflection of the health and religion relation, which is like a double-edged sword that needs to be balanced.

### Inconsistent or contradictory views

There were two main inconsistent or contradictory views within the 10 themes. First, regarding whether exemption from fasting was mandatory for participants with diabetes (theme 2), there were viewpoints of favoring, opposing, being neutral and reluctant to answer. In fact, due to several potential dangers and risks, religious regulations and medical recommendations both permitted exemption from fasting for participants with diabetes [49, 50]. Furthermore, given the different diabetes types and fasting-related complications, the American Diabetes Association (ADA) published the “Recommendations for management of diabetes during Ramadan” in 2005 [51] and its update in 2010 [50], in which participants with diabetes were classified into different risk groups with corresponding recommendations. Second, there are three opinions on physical activity (theme 7): decreasing movements to preserve energy, maintaining or increasing activity to reduce blood glucose, and opting to walk and refusing heavy exercise (neutral view). Previous studies recommend that participants should reduce physical activity, especially several hours before the sunset meal, to lower the risk of hypoglycemia, but this was mainly the experts’ opinion. Evidence from an empirical study provided concrete data that physical activity was not accountable for changes in glycemia [52]. In other words, there is no need to intentionally decrease movements. Moderate physical activity, such as walking, is a good choice for participants.

Therefore, as shown in category 4 (themes 8, 9, and 10), structured Ramadan-focused education is strongly recommended to address the abovementioned problems. HCPs and religious leaders can jointly organize education to direct Muslims with diabetes to fast safely, and HCPs and religious leaders have an opportunity to exchange information on religious culture and health care. It is beneficial to create a harmonious relationship between patients, HCPs, and religious leaders. Although several strategies and guidelines have been released for HCPs, their application and popularization are not far-ranging. These guidelines and strategies need to be strengthened.

### Strengths and limitations

This study only includes 11 published articles without retrieving the gray literature, so we cannot analyze unpublished relevant studies. The incoherence of the results of the included studies was not completely consistent, so we integrated information as extensively and accurately as possible to provide practical evidence. One minor detail about complications was reported in the 11 studies lacking the symptom of “thrombosis”, which was documented in several review articles [2, 3]. This notion indicates that data saturation may not be achieved in the 11 studies, the number of included studies may be insufficient, or this subject of qualitative studies requires further research. Regarding the types of diabetes, only T1DM and T2DM were reported within the included studies; thus, gestational diabetes mellitus and other specific types of diabetes were not applicable. In addition, the sample size of T1DM participants was small, so it was difficult to generalize the findings for all types of diabetes. It is self-evident that the heterogeneity of the participants is very high because they came from eight countries with different health states, various regional cultures, and different education backgrounds. Therefore, the summary of their subjective feelings may seem broad. In addition, all the authors of this study are non-Muslims; thus, we may lack deep religious empathy and experience difficulties in analyzing the information in a sufficient manner. However, since we are not believers of any religion, we can objectively and fairly analyze the information. Despite its limitations, the present study provides the first review and meta-synthesis of qualitative research on the subjective feelings of participants with T1DM and T2DM diabetes during Ramadan fasting.

## 5. Conclusions

In this meta-synthesis, we described and analyzed the extensive range of experiences and perspectives of people with diabetes observing fasting during Ramadan. Four categories and ten themes represent almost all of the aspects of Ramadan-related issues in participants with T1DM and T2DM diabetes.

Although the pathophysiology of T1DM and T2DM is different, they share many common experiences and views on Ramadan fasting itself. The exemption and reduction of medication for insulin oral medications in TIDM and T2DM are overlapped to some extent. The relationship between Muslims with diabetes and HCPs is actually a reflection of the health-religion relation, which is like a double-edged sword that needs to be balanced. The opinions on exemption from fasting for Muslims with diabetes are widely divided. Regarding the appropriate amount of physical activity for people with diabetes during Ramadan fasting, views vary.

The above-mentioned content further illustrates the importance and necessity of Ramadan-focused education for Muslims with diabetes. On one hand, the findings from this review highlight that the education is effective in decreasing health risks in participants with T1DM. The education program should be developed and generalized to Muslims with diabetes, at least for insulin-dependent participants. On the other hand, HCPs can join in the education program to provide supplemental information on the Islamic culture background and advantages of fasting. Therefore, HCPs are able to instruct their patients by weighing the health risks and mental satisfaction, partly, to balance health and religion.

Our study also sheds light on the psychological issues of Ramadan fasting in people with diabetes. They need help from all sectors of society, religious scholars or psychologists to dispel misgivings and doubts. The current study provides qualitative evidence and a preliminary direction for the development of intervention programs to optimize the management of Ramadan fasting for individuals with diabetes.

## Supporting information

S1 Checklist CASP systematic review-checklist. (PDF)

S2 Checklist PRISMA 2009 checklist-completed. (DOC)
